# Disaster Collaborative Exercises for Healthcare Teamwork in a Saudi Context

**DOI:** 10.1007/s13753-023-00484-z

**Published:** 2023-04-11

**Authors:** Mohammed Ali Salem Sultan, Amir Khorram-Manesh, Jarle Løwe Sørensen, Johan Berlin, Eric Carlström

**Affiliations:** 1 Model of Care, Healthcare Transformation, Regional Health Directorate, Najran, 66255 Saudi Arabia; 2 Institute of Health and Care Sciences, Sahlgrenska Academy, Gothenburg University, 405 30 Gothenburg, Sweden; 3 Institute of Clinical Sciences, Sahlgrenska Academy, Gothenburg University, 405 30 Gothenburg, Sweden; 4 Gothenburg Emergency Medicine Research Group (GEMREG), Sahlgrenska Academy, 413 45 Gothenburg, Sweden; 5 USN School of Business, University of South-Eastern Norway, 3199 Borre, Norway; 6 Department of Social and Behavioural Studies, University West, 461 86 Trolhättan, Sweden

**Keywords:** Collaboration exercises, Disaster education, Emergency management, Healthcare personnel training, Saudi Arabia, Teamwork

## Abstract

This study aimed to evaluate the development of healthcare teamwork during and after the collaboration tabletop exercises, through observation and interview methods. Integration and maturity theoretical models were employed to explain the collaborative challenges in teams that may suffer from unequally distributed power, hierarchies, and fragmentation. Using three-level collaboration tabletop exercises and the Command and control, Safety, Communication, Assessment, Treatment, Triage, Transport (CSCATTT) instrument, 100 healthcare workers were observed during each step in the implementation of the CSCATTT instrument using two simulated scenarios. The results show a lack of integration and team maturity among participants in the first scenario, leading to the delayed start of the activity, task distribution, and decision making. These shortcomings were improved in the second scenario. In-depth interviews with 20 participants in the second phase of the study revealed improved knowledge and practical skills, self-confidence, and ability in team building within trans-professional groups in the second scenario, which in concordance with the integration theory, was due to the attempts made in the first scenario. Additionally, there was an improvement in the team’s maturity, which in concordance with the maturity theory, was due to the knowledge and practical skills during scenario plays. These results indicate the importance of continuous tabletop training, and the use of CSCATTT as a collaborative instrument, to promote the development of collaboration and to test the concept of preparedness.

## Background

The escalating number of disasters and public health emergencies (DPHE) has led to a surge in property damages, deaths, and disabilities, and has overwhelmed healthcare services (Oktari et al. 2020) that requires “surge capacity” in a multi-agency approach, creating goal-oriented teams and partnerships to avoid the negative impacts of DPHEs (Khorram-Manesh 2020; OCHA 2022). According to the Merriam-Webster dictionary (2022), teamwork is used synonymously as collaboration and partnership but has attracted different definitions in disaster research (Dickinson and McIntyre 1997; Finn 2008; Xyrichis and Ream 2008; Finn et al. 2010). Finn (2008) defined teamwork as the negotiated outcome that brings the organization’s members to act, reason, behave, and work towards achieving a common organizational goal and objective (collaboration). Furthermore, according to Finn et al. (2010), teamwork is an action that involves uniting different groups of employees within an organization for a common identity and maximizing their inputs to attain management objectives. However, in practice, teams may have difficulties collaborating (Diefenbach and Sillince 2011) due to differences in cultures and the manifestation of factors such as comprehensive bureaucracies, decision hierarchies, language, goals and objectives, expectations in roles and responsibilities, and communication practices that may stem from distinct perceptions of what precisely a collaboration is and what it entails (Weick 1990; Grossman et al. 2021).

The World Health Organization (WHO) (2022) emphasized that teamwork is a cardinal prerequisite in all phases of emergency management. Poor collaboration during emergencies is a significant challenge affecting healthcare workers (HCWs) globally (Jamshidi et al. 2019). For instance, American HCWs, like those in Arabic countries, experience difficulties working together to minimize the complications of disasters (Schmutz et al. 2019; Alandijany et al. 2020), indicating a need to determine some strategies that governments and pertinent stakeholders in the health sector can utilize to foster teamwork and collaboration in DPHEs management (Gooding et al. 2022). It is common in countries such as Saudi Arabia (Robertson et al. 2002) to recruit healthcare staff from different countries, and the mix of cultural preferences may be an obstacle to communication (Ghalib 2019). In addition, Arabic countries are known to have a highly hierarchical organization (Hofstede 1984; Shackleton and Ali 1990; Robertson et al. 2002; Alamri et al. 2014) and power distance (PD), that is, the extent to which less powerful members of organizations and institutions accept and expect that power is distributed unequally (Hofstede 1991; Grossman et al. 2021). Organizational hierarchy and power distance may prevent team building by creating a time-consuming tradition of socializing (Ghalib 2019), which according to Aldulaimi (2019), prevents innovation and handling change due to inert tribal and clan traditions. Face-saving is common and may hinder distinctness (Yaseen 2010). There is also a strong preference for preserving group harmony and showing more respect for older leaders than younger and more competent participants (Mostafa 2003). Furthermore, women are often regarded as less capable than men to lead an organization at a high position (Mostafa 2003; Yaseen 2010; Ghalib 2019). Thus, based on current research and theory, there is a reason to expect that cultural and national diversities can impact the way one conceptualizes and approaches collaboration and that this influence may play out differently for collaborators, such as among employees in Saudi Arabia’s hospitals, hindering the effectiveness of teamwork (Alahmadi 2010).

Despite implementing strategies to promote and achieve efficient teamwork among their employees, many hospitals in Saudi Arabia struggle with factors such as the lack of accountability, poor decision making, and inadequate conflict management that challenge their efforts and prevent developing collaborating teams that effectively manage DPHEs (Sweis et al. 2013; Al Thobaity and Alshammari 2020; Sultan et al. 2020; Zajac et al. 2021). Against this background, the Saudi Health Ministry has launched multiple initiatives to improve teamwork among medical professionals (Moussa et al 2022). Nevertheless, collaborative activities are needed to adequately prepare HCWs to manage the ramifications of DPHEs (Alenazi et al. 2020) and to develop a team-building framework that involves all employees in a cross-disciplinary manner. Such activities, whether through massive online courses, webinars hybrid events, or video conferencing also help train and empower employees to solve current and prospective problems (Tanco et al. 2011; Sultan et al. 2021; Reiners and Jayhooni 2022), promoting safe and high-quality care (Rosen et al. 2018). This study aimed to evaluate the development of healthcare teamwork during collaboration exercises.

## Methods

This study used a mixed method approach, using three-level collaboration tabletop exercises combined with the Command and control, Safety, Communication, Assessment, Treatment, Triage, Transport (CSCATTT) instrument, a collaborative instrument originated from the Major Incident Medical Management and Support courses (MIMMS) (Sultan et al. 2021), to obtain quantitative data. The performance of 100 HCWs was assessed using the observational method during each step in the implementation of the CSCATTT instrument through two simulated scenarios. Finally, 20 HCWs were interviewed face-to-face to obtain qualitative data, as part of this mixed method approach.

### Course Design

A collaborative simulation exercise, using a collaborative instrument to exhibit stability and practice transitions, overlaps, seamlessness, and creative thinking, through a standardized pattern.

- The three-level collaboration (3LC) exercise is a validated model with a focus on collaboration, interagency participation, and joint decision making (Berlin and Carlström 2008). This model was chosen because it is one of the few models for collaboration exercises aimed at developing teams’ collaborative abilities and strengthening perceived levels of learning and utility by focusing on flexibility, improvisation, and joint evaluations and reducing organizational barriers (Khorram-Manesh, Berlin et al. 2016; Khorram-Manesh, Lupesco et al. 2016).
- The MIMMS stands for major incident medical management and support (Sammut et al. 2001) and encompasses CSCATTT, which presents the collaborative element, standing for command and control, safety, communications, assessment, triage, treatment, and transportation. The approach and standards recommended in CSCATTT have proven efficient and invaluable to military and civilian healthcare worldwide (McCormack and Coates 2015; Ronchi et al. 2016; Phattharapornjaroen et al. 2022).

### Conceptual Framework

This research employed two theoretical models to evaluate teamwork development and bridge the gap between knowledge and practice: (1) the integration of team members (Hall and Weaver 2001), and (2) team maturity (Sandberg 1997). These theoretical frameworks contribute to helping the HCWs integrate and collaborate in their work to overcome challenges (Makaram 1995). In addition, both frameworks support Petrie’s (1976) recommendation for “idea dominance,” which emphasizes that the members should be able to recognize their successes and achievements, individually and as part of the team. The choice of these classical frameworks is based on the characteristics of the organizational culture in Saudi. These frameworks explain not only idealistic teams but also the challenges of collaboration and change within a team in terms of degrees of integration and maturity.

The Hall and Weaver (2001) framework explains how the ability of teamwork brings various team members to work together to achieve a common goal. The interaction model highlights three main theoretical categories through which teamwork exists in an organization: multi-professional, interprofessional, and trans-professional. In a multi-professional team, every team member has a specialized role for which he or she is best suited (Hall and Weaver 2001). In an interprofessional team, every team member specializes in a specific role, but there are interactions between team members. In a trans-professional team, every team member has a specialized role, has the freedom to interact with other team players, and is prepared to act on behalf of any other team member when needed (Hall and Weaver 2001; Berlin et al. 2012). Such interactions require certain collaborative elements (Phattharapornjaroen et al. 2022). The Sandberg (1997) model of team maturity assesses the achievement of a team with organizational objectives. According to this model, teams can be immature, mature, or overripe. In contrast to a mature team, an immature team is characterized by a loose interconnection between team members, a subdivision within groups, and a high degree of individualism with no focus on the primary objective and the organization’s mission. An overripe team is rigid, with its team members lacking flexibility and excluding new members (Sandberg 1997). Therefore, the organization should strive to ensure that its team operates under the maturity category (Sandberg 1997; Berlin et al. 2012). Such maturity may be estimated using an instrument such as CSCATTT (Phattharapornjaroen et al. 2022).

The definition of collaboration in this study is based on teamwork’s integration and maturity, which define collaboration as a mix of activities and procedures shared among multiple levels (organizational and individual). It entails communication to yield shared responsibility and decision making (Castañer and Oliveira 2020) and creates interactivity by using a practical instrument like CSCATTT used as a planning and evaluation tool (Lozano et al. 2021). Integration and maturity are regarded as closely connected to collaboration, that is, an integrated and mature team will collaborate properly (Berlin 2014). In contrast, a non-integrated, immature, or overripen team may have severe difficulties in collaboration, and they will minimize their task-driven reciprocity and nurture a parallel and disintegrated behavior (Rogers and Mulford 1985; Whetten 1985; Berger and Luckman 1991). Berlin and Carlström (2008) have explained through a model the way team members correspond to organize and coordinate tasks in collaboration across three levels; the sequential method refers to the distribution of tasks by doing one after another in successive order, while with the parallel method team members from different disciplines meet and perform different tasks and activities simultaneously (Berlin and Carlström 2008). Finally, in the synchronous method, the team members come together to share the workload and accomplish tasks (Berlin 2010).

### Participants and Setting

One hundred HCWs from 10 hospitals belonging to the Ministry of Health in Najran, Saudi Arabia, were voluntarily enrolled in the 3LC training courses, held three times and on separate days with the same structure consisting of lectures and simulation scenarios, between October and November 2020. Najran is exposed to natural and human-made hazards, such as flash floods in wadis, fires, sandstorms, the Covid-19 pandemic, and armed conflicts (Saudi-Yemen Border). All staff engaged in disaster and emergency management in various positions and levels, who were present throughout the entire courses, were included. Other HCWs were excluded.

### Data Collection and Procedures

**Fig. 1:**
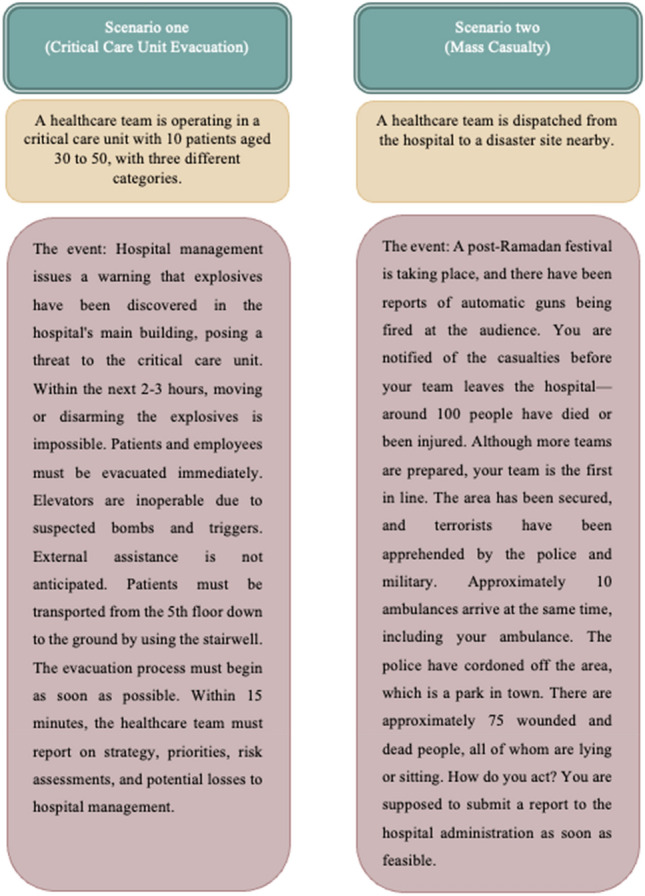
The two scenarios adopted from realistic events within a hospital context for 3LC exercise courses in Najran, Saudi Arabia, in October and November 2020

After theoretical lectures, the participants in the 3LC exercise courses were divided into four groups, consisting of physicians, nurses, and support services workers. They received the necessary instructions to complete a side-by-side exercise in two simulated scenarios. In these micro-exercises, CSCATTT was used to enable the standardized management of DPHE. Scenarios were adopted from two realistic events within a hospital context (Fig. 1). The management time started when the scenario was presented by the exercise leaders during each scenario exercise. After the completion of the allotted time for working on the scenario, each team demonstrated how it tackled the situation on its own. The exercise leaders then gave the participants a rapid recap of the scenario. Following that, each table’s attendees were asked to consider what they could have done differently if the same scenario was presented once again. Everything that participants did in the scenarios and their performances was observed and registered by the exercise leaders at each team table.

#### Approach 1: Observational

During the 3LC simulation training, a participant observation model was used to monitor each group’s performance and practical progress between the two scenarios by one observer, with a focus on time, and the 13 elements of CSCATTT, including appointing a leader, measuring the time to set up the organization, achieving a consensus in the team, distributing tasks among team members, prioritizing activities, identifying external resources, assessing the progress of mission (making new decisions or end the mission), evaluating the progress of the exercise (need for new inputs after discussion with exercise leaders or cancelling the exercise), time to the main decision, time to triage, time to treatment, time to documentation, and time to report to senior management. After each scenario exercise, the performance and practical progress of the participants were reviewed and discussed by the exercise leaders to reach a consensus. During the scenario play, the observer noted whether the participants followed the CSCATTT elements by identifying a commander and whether the commander managed the teamwork.

The observer also noted whether the commander ensured successful incident management, that is, whether he/she assembled a team of multiple personnel capable of overcoming a potentially chaotic scenario and whether the team advanced to the sub-tactical, tactical, or operational levels. The data were collected via the event sampling technique (Black 1996), which allowed for emphasizing one specific subject. All other forms of conduct were dismissed. A short hot wash-up discussion was conducted after each scenario exercise. These discussions sought to address the observational data under each scenario, in addition to confirming and discussing the outcomes and improvement measures.

#### Approach 2: Interviews

The lead author of this article conducted individual semi structured interviews (mainly face-to-face, in-depth interviews using open-ended questions) on a systematic basis (Holloway and Galvin 2017) with voluntary HCWs, enrolled in 3LC courses. The two theoretical models—integration of team members in the team and team maturity—were explained and described to the interviewees during the interviews. The sampling was according to Holloway and Galvin’s (2017) recommendation, that is, purposive or convenience sampling. This means a selection of participants who were expected to provide the best information. The method of introducing a theoretical framework before the interview aimed to motivate the respondents to interpret their experiences in terms of the presented concepts (Larsson et al. 2017; Wennman et al. 2019). Out of the 100 participants who were emailed by a medical secretary in each hospital, 34 agreed to participate. Interviews were scheduled in the participants’ work environments and during regular work. Saturation was reached (repetition, recognition, and reduced variation in answers) with 20 responders and the interviews were finalized (Morse 2012).

The participants were informed that participation was voluntary and had no bearing on their job grades. They were asked to read and sign an informed consent form before their interviews. The lead author piloted the interview questions before the principal data-gathering point. The pilot study involved three staff members (physician, nurse, and paramedic) working in critical care settings. These respondents were not included in the main study. There were no revisions needed in the interview guide after the pilot study. The interview guide was in English and included the demographic data and 10 questions. The participant’s initial answers allowed obtaining a comprehensive view of their experiences related to exercises within the scope and goal of the study (Brinkmann and Kvale 2015). Each interview lasted 50–60 min, and the audio was recorded and transcribed verbatim. The lead author used a field note and a reflective journal to record the learning-related incidents and to ponder the answers provided by the participants during the data collection period.

### Data Analysis

Microsoft Excel was used to analyze quantitative data in the observational approach and the interview data were analyzed by directed content analysis suggested by Hsieh and Shannon (2005). The strength of a directed approach to content analysis is its ability to support and expand a theoretical framework, which aims to conceptually extend a framework and help the authors focus on the research question (Hsieh and Shannon 2005). Based on distinctions in the role of theory, Potter and Levine-Donnerstein (1999) suggested categorization as a deductive analysis. In the early stages of the analysis, we focused on gaining a more comprehensive understanding of the data regarding the research phenomena, which is the condition for teamwork building among HCWs in Saudi Arabia with the intent of handling DPHEs.

## Findings

Of the 100 participants (37 females and 63 males), 82% were Saudi nationals and 18% were multinational. Around 27% of the participants were under 29 years while 19% were over 40, and the most common age group aged between 30 and 39 (54%). Around 37% of the respondents were nurses. The experience levels of the participants ranged from around 5 years to over 25 years and 8% had more than 21 years of experience (Table 1).

**Table 1:** Demographic data of the observational approach (*n* = 100)

Variable	*n*	Variable	*n*
*Age by year*		*Position*	
20–29	27	Supportive Services (SR)	11
30–39	54	Physician (DR)	32
40–54	15	Nurse	
55+	4	(NR)	37
		Paramedic (PR)	12
		Hospital Director (HD)	8
*Gender*		*Level of education*	
		Diploma	12
Male	63	Bachelor	71
Female	37	Master’s	14
		Ph.D.	3
*Experience by year*		*Nationality*	
1−9	57	Saudi	82
10−20	35	Non-Saudi	18
21−35	8		

### Observational Findings

#### Scenario 1

In this scenario, there was a lack of integration among the team members, leading to delayed preparation and the start of the work. There was difficulty understanding the scenario content, which required further clarification by the exercise leaders. Most of the team members were aware of the need to use reversed triage principles during the event. Following the CSCATTT strategy, the distribution of roles was clear, and the managers focused on logistics and communication; the physicians focused on treatment, triage, and equipment; and the nurses focused on collaborating with physicians on triage and treatment, securing continuity, and giving patients comfort. Others were prepared to assist during heavy evacuation work. The teams worked on the scenario, but the interaction was intermittent and hesitant.

In the second round, the team leaders pointed out the need for the following improvements: developing protocols for follow-up, for example, action cards for all involved staff, multiagency inclusion in the disaster plans, plans for internal coordination of the hospital’s departments, using a unified model for triage of patients in time- and staff-constrained circumstances, and plans for operating reversed triage. Furthermore, it was suggested to develop drills to enhance the understanding of role distribution. The time for decision making was long (median = 18 min), with one exception, the appointment of a leader, which ranged between 3 and 8 min (median = 4 min). Some of the tabletop exercise leaders’ comments are as follows:

The teams followed the CSCATT elements, but some of them were not sure whether they were on the right track. Some were confused about where to start and how to distribute the mission. (Tabletop exercise leader, team B)

There was good communication, but it took some time to distribute the task, and the team was excellent at using the CSCATTT model. (Tabletop exercise leader, team D)

Overall, professional performance during a difficult and time-constrained situation involved ethical dilemmas and severe logistic challenges. The teams handled the situation very well, but new exercises to measure the outcomes would be helpful. (Tabletop exercise leader, HA)

#### Scenario 2

In this scenario, all groups improved their work, and using the CSCATTT instrument, they appointed the team leader timely, tasks and responsibilities were rapidly distributed, and decision making was quicker. This time, the participants were aware of different needs and, therefore, if necessary, ready to manage the situation across professions and organizations. Collaboration within the teams improved from sequential and parallel to synchronous approaches. Moreover, there was a noticeable improvement in the teams’ performance regarding the medical and non-medical responses and triage of patients. The participants gained more awareness of what was required of them and how to become organized.

In the second round, the team leaders pointed out the following potential improvements: preparing safety and primary and secondary triage zones, establishing a treatment strategy, coordinating with military and police forces, designing entrance and exit for ambulances, prioritizing security, and performing dynamic triage. The issues discussed with teams during the second round included the following: (1) disagreements between the teams about the prioritization of patients during mass causalities; (2) practice of reversed triage (time to and performance); (3) time-critical events in terms of expectancy and efficiency; (4) collaboration with other organizations; (5) possibility of temporary integration of police and military staff as part of the healthcare team; (6) using the ambulance staff at the scene. The time to decide was shorter among the teams, which may indicate coherence and preparedness. Nevertheless, there was one exception, the time to set up an organization, which took 9–11 min (median = 10 min) (Table 2). The exercise leader’s observations based on CSCATTT showed improved results compared with Scenario 1. The following are some comments from the exercise leaders:

The team improved remarkably in following CSCATTT elements compared to Scenario 1. This time, they started to document a hospital report from the start, and it was sent as soon as possible. (Tabletop exercise leader, HSA)

They started to think logically and report to senior management as soon as they figured out the disaster. They applied CSCATTT directly this time. Again, overall, the teams showed satisfactory performance during difficult and time-constraining situations involving ethical dilemmas and severe logistic challenges. (Tabletop exercise leader, MJ)

**Table 2:** Participants’ observation model of performance and practical progress between the two scenarios with a focus on time (*n* = 100)

Variable	Scenario 1 (Median values)	Scenario 2 (Median values)
Leader appointed	4	2
Consensus achieved in the team	15	14
Tasks distributed in the team	6	4
Common activities prioritized	15	12
External resources identified	15	11
Mission completed	33	30
Exercise cancelled	34	30
Time to set up an organization	10	10
Time to the main decision	18	12
Time to triage	19	8
Time to treatment	26	16
Time to documentation	28	26
Time to report to senior management	27	17

### Findings of the Interviews

The results of semistructured interviews with 20 of the HCWs, which show various perceptions, were divided into four themes: overall impression and improvement of performance; multi-, inter-, and trans-professional teamwork and collaboration; immature, mature, and overripe team dynamic; and future development and vision of Saudi Arabian emergency response and preparedness. The HCWs reported a high level of improvement in knowledge and practice, skills, confidence, and team building following the implementation and hot wash-up discussion after each scenario. HCWs indicated that collaboration exercises using the CSCATTT instrument were crucial for developing their knowledge and practice and could help them prepare for disasters and unforeseen events.

The participants reported that the trans-professional team developed because they understood the scenarios. They underscored that they developed their skills and knowledge of disaster management because of the attempts they made in Scenario 1. On the theoretical framework of team maturity by Sandberg (1997), several participants indicated that immaturity in Scenario 1 was due to the team members not having the same knowledge and skills. While in Scenario 2, they become mature because of gaining knowledge, training, and resolving challenges that result in individual and team development. Overripe team members sought control over every aspect and showed less flexibility but in the end, the rest of the team took control of the situation, and with no disagreement. Moving into the future, the HCWs reported that the 3LC exercise is vital for collaboration and CSCATTT is a necessary instrument for evaluation of the outcomes in disaster management.

## Discussion

This study emphasized the impact of culture, knowledge, and skills in the management of DPHEs and the importance of simulation training and evaluation tools for collaboration improvement and assessment of such progress.

### Impact of the Collaboration Exercises

In this study, the use of 3LC as a collaborative exercise and CSCATTT as a collaborative tool improved participants’ knowledge, skills, integration and collaboration, and confidence, through repeated exercise. According to Al-Hunaishi et al. (2019), disaster training may improve self-efficacy and increase willingness to participate and work in DPHEs. Although the HCWs were not confident to act in Scenario 1 of this study, they understood the necessary steps of action in Scenario 2. Thus, continuous training can increase HCWs’ knowledge and efficiency, thus their confidence in handling victims of unexpected DPHEs (Sultan et al. 2020; Bistaraki et al. 2011; Cariaso-Sugay et al. 2021). Although there is disagreement among scholars over the meaning of trust, most practitioners and scholars concur that there is a connection between trust and collaboration (Ross and LaCroix 1996; Dirks and Ferrin 2001) and that trust is one of the key requirements for effective collaboration (Kouzes and Posner 2003). Continuous training may increase HCWs’ trust and willingness to work in cross-sectoral capacities to handle DPHEs (Burnett and McGuire 2020). The combination of 3LC and the CSCATTT instrument has recently illustrated improvement in role distributions, enhancing the professional thinking in the field to respond to DPHEs by strengthening staff confidence, and lessening their internal fear of making mistakes (Sammut et al. 2001; Phattharapornjaroen et al. 2020). The exercise leaders’ observations based on CSCATTT showed special attention to collaboration development (Borell and Eriksson 2013), regarding all elements of the instrument to guarantee command and control formation, role identification, and the assessment of short- and long-term consequences in planning and management of DPHEs. The exercise also offered multiagency discussions and learning opportunities from the mistakes in a safe environment that offers the trial of new tactics, establishing a sense of security and order across sectors (Andersson et al. 2013). The distribution of tasks based on CSCATTT reduced hierarchical communication, coordination, and collaboration, and obstacles and ambiguity in the team (Sammut et al. 2001), as reported by Phattharapornjaroen et al. (2020) who emphasized that CSCATTT as a collaborative instrument minimizes organizational barriers in favor of a collective increase in skills and reduction in limitations, and allows transition between various strategies needed, depending on the situation and leadership types (Sammut et al. 2001; Kahn et al. 2017).

### Teamwork Integration and Maturity

Exercises based on the trans-professional approach to DPHE management enable HCWs to work under different conditions, adapting to the dynamics of disasters (Al-Hunaishi et al. 2019; Melin Emilsson et al. 2020) and learning to work towards a unanimous goal. Developing team bonds, they can work as a team in severe conditions and understand each other’s capabilities and limitations (Sasangohar et al. 2020). This transition was seen in this study between the first and second scenarios.

The concept of immature behavior in this study referred to HCWs with inadequate knowledge and skills (Miller et al. 2018). However, as they were trained, they acquired relevant knowledge and skills, as demonstrated in Scenarios 1 and 2, when the immaturity of most HCWs vanished and almost every one of them was mature enough to take part in the event at the end of Scenario 2. Repeating scenarios can help the team members move from immaturity to maturity without becoming overripe, and simultaneously avoid overripe team members, who do not fit into the team due to tribal and clan traditions and other factors that influence teamwork (Sandberg 1997; Joseph 2000; Yaseen 2010; Tlaiss and Kauser 2020). However, according to Hathaway et al. (2022), more research may point out remedies for developing mixed teams of former overripe members to become mature. It is also important to bear in mind that one traditional behavior, which shows a preference for preserving group harmony, may be successful during teamwork in some situations, while it could be detrimental in other situations (Weick 1990; Aldulaimi 2019).

### Saudi Culture and Awareness

Healthcare workers need to understand how their traditions and beliefs may positively impact teamwork and collaboration in emergency management based on the CSCATTT strategy (Phattharapornjaroen et al. 2020; Lozano et al. 2021). According to this study’s findings, although some team members were overripe or immature, others were mature and ready to work as a team. As Abu Alsuood and Youde (2018) have observed, Saudi culture emphasizes the need for leaders to help their followers prioritize continuous improvement. Leaders are also typically expected to guide their followers instead of relying on an instructional approach. These traditions are likely to affect how teams in the health sector operate, including those that manage emergencies. Therefore, efforts to promote collaboration and teamwork in emergency management should consider how traditions associated with Saudi culture influence teams operating in the health sector (Assbeihat 2016). Continuous training allows HCWs to share ideas, knowledge, and skills in disaster management (Buljac-Samardzic et al. 2020).

The future of disaster management relies on the awareness of HCWs and public members. More scenarios and hot wash-up seminars will create more awareness about collaboration among HCWs and enable them to work efficiently and manage the DPHEs (Carpenter 1995). This study, like previous studies, has confirmed that collaboration exercises in disaster management contribute to a sufficient degree of the knowledge and skills needed for healthcare teams in their preparedness, self-efficacy, confidence, and willingness to work (Berlin and Carlström 2015; Khorram-Manesh, Berlin et al. 2016; Khorram-Manesh, Lupesco et al. 2016; Calovi 2018; Magnussen et al. 2018; Sørensen et al. 2019; Simon et al. 2021). In addition to training HCWs, public awareness of disaster management in how to mitigate the risks for DPHEs can improve the management system in Saudi Arabia. To educate the public to act at various levels of DPHEs management or even to raise their awareness of any event to lessen the negative effects of any incidents, the CSCATTT elements can be employed (Khorram-Manesh et al. 2017; Khorram-Manesh 2019; Glantz et al. 2020). Public campaigns can be conducted in schools, markets, religious venues, and other public places to help the public understand and prepare for their role in the management of DPHEs globally.

### Limitations

The first limitation of this study is the sample size (Noyes et al. 2018). However, the results may indicate a trend and pave the way for future research. Second, the approach selected for this study could be biased because of the involved researchers’ preferences. Hence, evidence supporting a theory may be easier to get across than evidence refuting it (Ruggiano and Perry 2019). Third, the interview responses by HCWs could also be biased depending on their attitude and view of the subject matter and the persuading act of the interviewer (Hsieh and Shannon 2005). Finally, how the data are collected can affect their accuracy (Sherif 2018).

## Conclusions

This study focused on evaluating the development of healthcare teamwork during collaborative exercises by exposing HCWs to two scenarios, using a collaborative exercise model and a collaborative instrument, based on two theoretical frameworks. The tabletop training enabled the integration of the teams and maturity in team building and disaster management. The use of the CSCATTT instrument contributed to promoting the development of collaboration, communication, and coordination among the team members and allowed transition between various strategies as needed depending on the situation. The theoretical frameworks used contributed to explaining changes and development within a team in terms of integration and maturity. In-depth interviews revealed improved knowledge and practical skills, self-confidence, and ability in team building at the end of the study, which in concordance with the integration theory, was due to the attempts made in the first scenario. Additionally, the improvement in the team’s maturity, in concordance with the maturity theory, was due to the gained knowledge and practical skills during scenario plays. These results indicate the importance of continuous tabletop training, and the use of CSCATTT as a collaborative instrument, among all response agencies in Saudi Arabia, to promote the development of collaboration and to test the concept of preparedness. The study results also indicate a need to incorporate collaborative tabletop exercises in existing education and training curricula, among all involved disaster response agencies.
